# Risk of post-stroke pneumonia with proton pump inhibitors, H_2_ receptor antagonists and mucoprotective agents: A retrospective nationwide cohort study

**DOI:** 10.1371/journal.pone.0216750

**Published:** 2019-05-08

**Authors:** Tae-Jin Song, Jinkwon Kim

**Affiliations:** 1 Department of Neurology, College of Medicine, Ewha Womans University, Seoul, Korea; 2 Department of Neurology, Gangnam Severance Hospital, Yonsei University College of Medicine, Seoul, Korea; 3 Department of Neurology, CHA Bundang Medical Center, CHA University, Seongnam, Korea; University of Ioannina School of Medicine, GREECE

## Abstract

Stroke patients are at high risk of developing pneumonia, which is major cause of post-stroke mortality. Proton pump inhibitors and H_2_ receptor antagonists are anti-ulcer drugs, which may predispose to the development of pneumonia by suppression of the gastric acid with bactericidal activity. Unlike proton pump inhibitors and H_2_ receptor antagonists, mucoprotective agents have gastroprotective effects with no or less anti-acid property. We aimed to investigate effects of the acid-suppressive medications (proton pump inhibitors and H_2_ receptor antagonists) and mucoprotective agents on risk for post-stroke pneumonia using the National Health Insurance Service-National Sample Cohort in Korea. This retrospective cohort study included 8,319 patients with acute ischemic stroke. Use of proton pump inhibitors, H_2_ receptor antagonists, and mucoprotective agents (rebamipide, teprenone, irsogladine, ecabet, polaprezinc, sofalcone, sucralfate, and misoprostol) after stroke were determined based on the prescription records, which were treated as time-dependent variables. Primary outcome was the development of post-stroke pneumonia. During the mean follow-up period of 3.95 years after stroke, 2,035 (24.5%) patients had pneumonia. In the multivariate time-dependent Cox regression analyses (adjusted hazard ratio [95% confidence interval]), there was significantly increased risk for pneumonia with use of proton pump inhibitors (1.56 [1.24–1.96]) and H_2_ receptor antagonists (1.40 [1.25–1.58]). In contrast to the proton pump inhibitors and H_2_ receptor antagonists, use of mucoprotective agents did not significantly increase the risk for pneumonia (0.89 [0.78–1.01]). In conclusion, the treatment with proton pump inhibitors and H_2_ receptor antagonists was associated with increased risk for pneumonia in stroke patients. Clinicians should use caution in prescribing the acid-suppressive medications for the stroke patients at great risk for pneumonia.

## Introduction

Stroke is the leading cause of death and long-term disability worldwide [1]. Stroke victims frequently have aspiration events and coexisting comorbidity such as old age, diabetes mellitus (DM), malnutrition and physical inactivity, which are well-established risk factors for infection and pneumonia [2]. Pneumonia is the most frequent post-stroke infection which constitute a leading cause of early and long-term mortality and morbidity after stroke [3, 4]. Therefore, identifying risk factors for pneumonia is important in prevention of the complication and improving long-term outcome after stroke. In stroke patients, gastric acid suppressive medications of proton pump inhibitors (PPI) and H_2_ receptor antagonists (H2RA) are frequently prescribed to control heart-burn symptom or prevent gastroduodenal injury. Growing evidence suggests that the acid-suppressive medications may increase risk of pneumonia by attenuation of the bactericidal effect of gastric acid [5, 6]. There were some prior researches for association between pneumonia and exposure to the PPI and H2RA during acute period of stroke [7–9]. However, there is insufficient data for the relationship between the risk for post-stroke pneumonia and the medications during long-term follow-up period. Beside PPI and H2RA, there are another types of anti-ulcer drugs called mucoprotective agents (rebamipide, teprenone, irsogladine, ecabet, polaprezinc, sofalcone, sucralfate, and misoprostol) with no or less anti-acid property [10]. Without gastric acid suppression, their effects on post-stroke pneumonia might be different to PPI and H2RA. To evaluate effects of the anti-ulcer drugs on the risk for post-stroke pneumonia, we conducted a retrospective cohort study using the nation-wide health insurance database which contained long-term data for the development of pneumonia and prescription records.

## Materials and methods

### Data sources

This was a retrospective cohort study using the nationwide population-based sample cohort by the National Health Insurance Service in Korea (NHIS-NSC) [11]. NHIS-NSC was constructed with 1,025,340 participants sampled randomly and stratified by sex, age, and household income, who were approximately 2.2% of the total eligible Korean population in 2002. Because NHIS is a single-payer program in Korea, NHIS-NSC contained whole health insurance claims data including hospital visits, procedures, diagnosis, prescriptions and demographic information of sex, age, household income, and death statistics of the included subjects. At each hospital visit diagnostic codes were recorded according to the International Statistical Classification of Diseases, 10^th^ revision (ICD-10). Requests for access to NHIS data can be made through the homepage of National Health Insurance Sharing Service [http://nhiss.nhis.or.kr/bd/ab/bdaba021eng.do]. To gain access to the data, a completed application form, a research proposal and the applicant’s approval document from the institutional review board should be submitted to and reviewed by the inquiry committee of research support in NHIS. The NHIS-NSC data were fully anonymized and did not contain any identifiable information. This study was approved, and informed consent was waived by the Institutional Review Board of Bundang CHA Medical Center (2017-08-047).

### Study subjects and outcome

**Fig 1:**
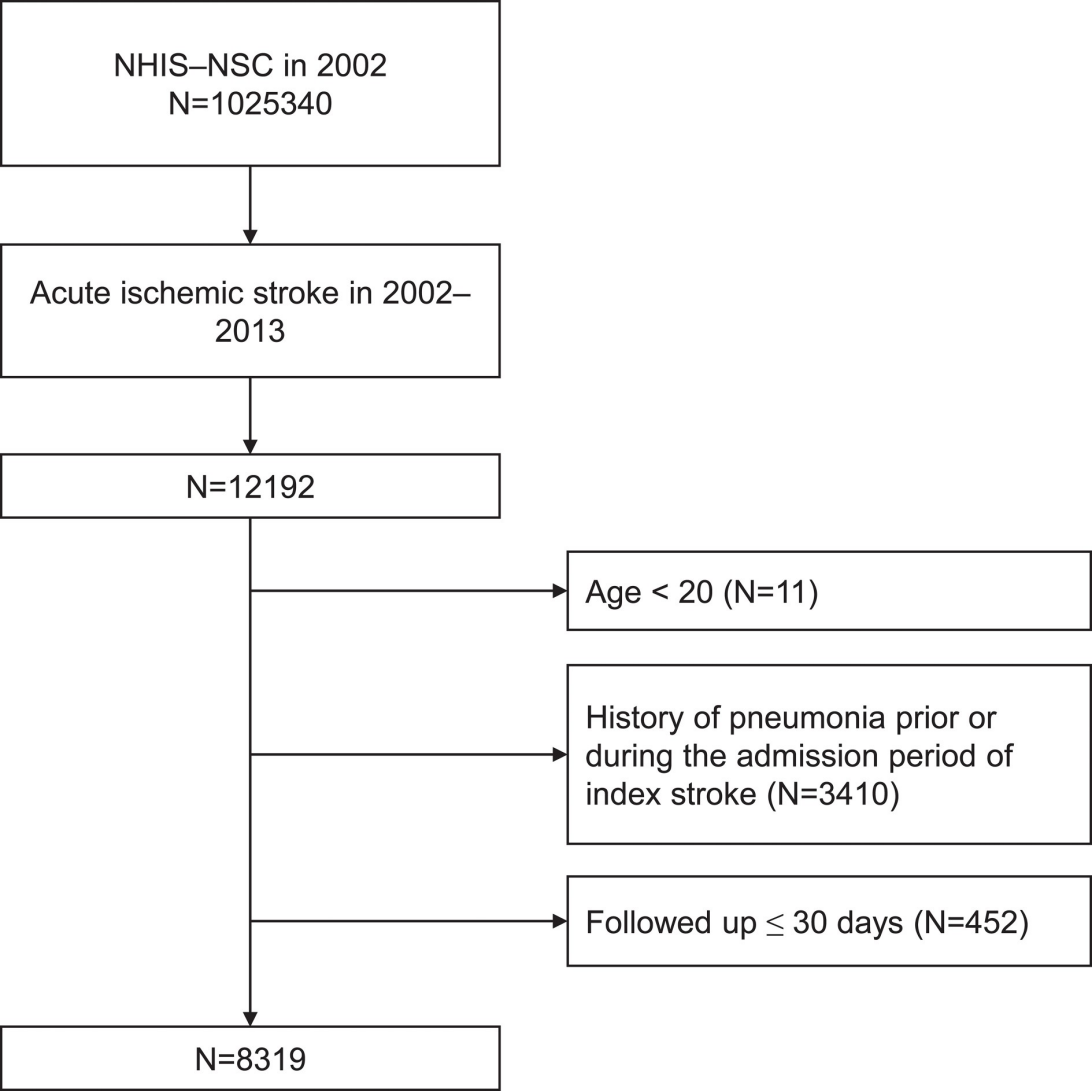
Flowchart of included patients.

We included patients aged ≥ 20 years who hospitalized (admitted or visited emergent medical center) with primary diagnosis of ischemic stroke (ICD–10 code of ‘I63’) between 2002 and 2013. To include only acute ischemic stroke patients, we selected patients who underwent brain computed tomography or magnetic resonance imaging during hospitalization due to the assumption that patients with acute stroke should undergo brain imaging [12]. Primary outcome is time to development of pneumonia after discharge which is determined based on the presence of diagnostic codes ‘J10–J18’[13–15]. To detect only patients who newly developed pneumonia after stroke as outcome, we excluded patients who had diagnostic codes ‘J10–J18’ prior to admission or within a month after discharge of index stroke. Flowchart for inclusion and exclusion criteria is shown in Fig 1. The included patients were followed up until the development of pneumonia, loss of participant eligibility for NHIS, or December 2013.

### Collection of prescription records

NHIS-NSC contained prescription data including drug name, dose, duration and date of prescription. We collected prescription records of included patients for PPI (omeprazole, pantoprazole, lansoprazole, rabeprazole, esomeprazole, and dexlansoprazole), H2RA (ilaprazole, cimetidine, famotidine, lafutidine, nizatidine, ranitidine, and roxatidine), and mucoprotective agents (rebamipide, teprenone, irsogladine, ecabet, polaprezinc, sofalcone, sucralfate, and misoprostol) which were available in Korea during study period. Based on the prescription records, exposure to PPI, H2RA, and mucoprotective agents were accessed on every day of the follow-up period, which were collected as time-dependent variables. According to the daily dose of PPI and H2RA, they were classified into ‘low dose’ and ‘high dose’. ‘High dose’ is determined if daily dose is ≥ 20 mg of omeprazole, 40mg of pantoprazole, 30mg of lansoprazole, 20mg rabeprazole, 30mg of esomeprazole, 30mg of dexlansoprazole, 20mg of ilaprazole, 800mg of cimetidine, 40mg of famotidine, 20mg of lafutidine, 300mg of nizatidine, 300mg of ranitidine, and 150mg of roxatidine; the cut-off values are *defined daily dose* determined by the World Health Organization [16].

### Covariates

We collected data for baseline characteristics of sex, age, household income at index stroke and the presence of underlying risk factors of hypertension, DM, myocardial infarction (MI), atrial fibrillation (AF), and chronic obstructive pulmonary disease (COPD). In NHIS-NSC, age was grouped into 5-year periods, which was treated as a continuous variable in the analysis. We stratified the household income level into tertile groups (low, middle, and high). The history of hypertension (I10–15), DM (E08–11, E13–14), MI (I21), AF (I48), and COPD (J42, J43 [except for J43.0], J44) were determined by the presence of diagnostic code before or during admission period of index stroke [12,17,18]. Hypertension and DM were recognized as relevant only if the subjects received one or more prescriptions of antihypertensive or antidiabetic medications with the diagnosis [19]. Use of thrombolysis (653500660, 653500670 for intra-venous and M6631, M6632, M6633 for intra-arterial) was determined by the procedure codes. We accessed length of hospital stay; patients were grouped into two groups (≤ 17 days, > 17 days) by the median length of hospital stay at index stroke. Hospitals at index stroke were classified into ‘general hospital’ and ‘hospital or clinic’ based on the healthcare resources (the former being a large-scale hospital). Years of index stroke were grouped according to the following: [2002–2005], [2006–2009], and [2010–2013].

### Statistical analyses

Use of PPI, H2RA, and mucoprotective agents though follow-up period were treated as time-dependent variables for the statistical analyses. Plots for estimated pneumonia-free probability according to the use of medications during follow-up are illustrated using the method established by Simon and Makuch [20]. The hazard ratio (HR) and 95% confidence interval (CI) were derived from time-dependent Cox proportional hazard regression models. The assumption of proportional hazards of the Cox models was tested by evaluating scaled Schonfeld residuals, which were found to be satisfactory. To identify independent risk factors for pneumonia, adjustments were performed for sex, age (as continuous variable), history of hypertension, DM, MI, AF, COPD, use of thrombolysis, household income, length of hospital stay, hospital type and year of index stroke. The data manipulation and statistical analyses were performed with PostgreSQL, version 10.1 (The PostgreSQL Global Development Group; https://www.postgresql.org/), and R software, version 3.4.4 (The R Foundation for Statistical Computing, Vienna, Austria; http://www.R-project.org/). A two-sided p value of <0.05 was considered to be statistically significant.

## Results

### Study subjects

**Fig 2:**
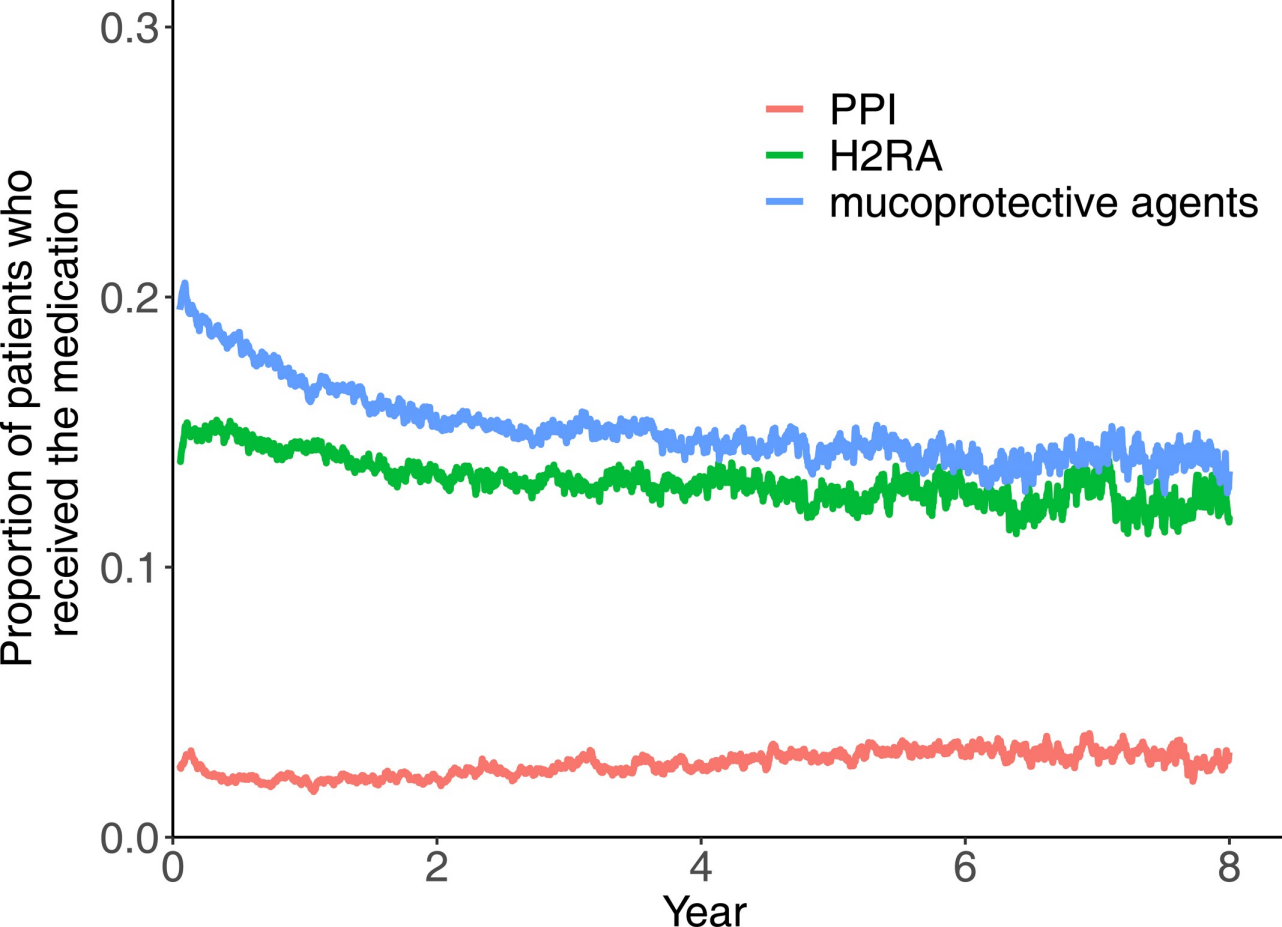
Proportion of patients who received medications thoughout the post-stroke period. Mucoprotective agents include rebamipide, teprenone, irsogladine, ecabet, polaprezinc, sofalcone, sucralfate, and misoprostol. Abbreviations: H_2_RA, H_2_-receptor antagonist; PPI, proton-pump inhibitor.

This study finally included 8,319 patients of acute ischemic stroke who discharged without pneumonia (Fig 1). The median age group was 65–69 [interquartile rage, 55–59; 75–79] years old, and males were 54.7% (Table 1). Fig 2 demonstrates the proportions of patients who received PPI, H2RA, and mucoprotective agents throughout the follow-up period after stroke. The proportion of patients received the medications were relatively consistent during follow-up. At 1-year after stroke discharge, there were 6710 patients who remained at risk for pneumonia (they survived and had no pneumonia until the time point). Among the 6710 patients at 1-year, the number of patients who received PPI, H2RA, mucoprotective agents at the time were 147 (2.2%), 971 (14.5%), and 1131 (16.9%), respectively. Table 2 shows the difference in characteristics of patients who received and those who did not receive the medications at 1-year.

**Table 1:** Baseline characteristics of included patients.

Variable	N (%) or median [interquartile range]
Total number of patients	N = 8319
Sex, male	4551 (54.8)
Age, year	65–69 [55–59; 75–79]
Hypertension	6038 (72.6)
Diabetes mellitus	2408 (28.9)
Myocardial infarction	610 (7.3)
Atrial fibrillation	811 (9.7)
Chronic obstructive pulmonary disease	1705 (20.5)
Thrombolysis (intravenous or intraarterial)	187 (2.2)
Household income	
low	2730 (32.8)
middle	2993 (36.0)
high	2596 (31.2)
Hospital type	
general hospital	7017 (84.3)
hospital or clinic	1302 (15.7)
Length of hospital stay	
≤17 days	4491(51.6)
>17 days	4028 (48.4)
Year of index stroke	
2002–2005	2392 (28.8)
2006–2009	3168 (38.1)
2010–2013	2759 (33.2)

Data are number (%) or median [interquartile range].

**Table 2:** Clinical characteristics of patients according to current use of medications at 1-year after index stroke.

Medication	Proton pump inhibitor			H2-receptor antagonist			mucoprotective agent*		
Variable	No use (N = 6563)	Use (N = 147)	P value	No use (N = 5739)	Use (N = 971)	P value	No use (N = 5579)	Use (N = 1131)	P value
Sex, male	3580 (54.5)	85 (57.8)	.481	3183 (55.5)	482 (49.6)	.001	3037 (54.4)	628 (55.5)	.523
Age, year	65–69 [55–59; 70–74]	65–69 [55–59; 75–79]		65–69 [55–59; 70–74]	65–69 [55–59; 75–79]		65–69 [55–59; 70–74]	65–69 [55–59; 70–74]	
Hypertension	4661 (71.0)	116 (78.9)	.046	4020 (70.0)	757 (78.0)	< .001	3930 (70.4)	847 (74.9)	.003
Diabetes mellitus	1789 (27.3)	46 (31.3)	.321	1538 (26.8)	297 (30.6)	.016	1487 (26.7)	348 (30.8)	.005
Myocardial infarction	459 (7.0)	10 (6.8)	< .001	403 (7.0)	66 (6.8)	0.852	383 (6.9)	86 (7.6)	.410
Atrial fibrillation	567 (8.6)	16 (10.9)	.419	498 (8.7)	85 (8.8)	.987	469 (8.4)	114 (10.1)	.078
Chronic obstructive pulmonary disease	1218 (18.6)	49 (33.3)	< .001	1046 (18.2)	221 (22.8)	.001	1026 (18.4)	241 (21.3)	.025
Thrombolysis (intravenous or intraarterial)	122 (1.9)	2 (1.4)	< .001	92 (1.6)	32 (3.3)	< .001	90 (1.6)	34 (3.0)	.002
Household income			.983			.105			.724
low	2112 (32.2)	48 (32.7)		1821 (31.7)	339 (34.9)		1803 (32.3)	357 (31.6)	
middle	2370 (36.1)	52 (35.4)		2076 (36.2)	346 (35.6)		2002 (35.9)	420 (37.1)	
high	2081 (31.7)	47 (32.0)		1842 (32.1)	286 (29.5)		1774 (31.8)	354 (31.3)	
Hospital type			.506			.398			.001
general hospital	5561 (84.7)	128 (87.1)		4875 (84.9)	814 (83.8)		4693 (84.1)	996 (88.1)	
hospital or clinic	1002 (15.3)	19 (12.9)		864 (15.1)	157 (16.2)		886 (15.9)	135 (11.9)	
Length of hospital stay			.803			.008			< .001
≤17 days	3436 (52.4)	79 (53.7)		3045 (53.1)	470 (48.4)		2996 (53.7)	519 (45.9)	
>17 days	3127 (47.6)	68 (46.3)		2694 (46.9)	501 (51.6)		2583 (46.3)	612 (54.1)	
Year of index stroke			< .001			< .001			< .001
2002–2005	2093 (31.9)	9 (6.1)		1862 (32.4)	240 (24.7)		1890 (33.9)	212 (18.7)	
2006–2009	2707 (41.2)	54 (36.7)		2396 (41.7)	365 (37.6)		2278 (40.8)	483 (42.7)	
2010–2013	1763 (26.9)	84 (57.1)		1481 (25.8)	366 (37.7)		1411 (25.3)	436 (38.5)	

*rebamipide, teprenone, irsogladine, ecabet sodium, polaprezinc, sofalcone, sucralfate, and misoprostol.

### Risk for pneumonia

**Fig 3:**
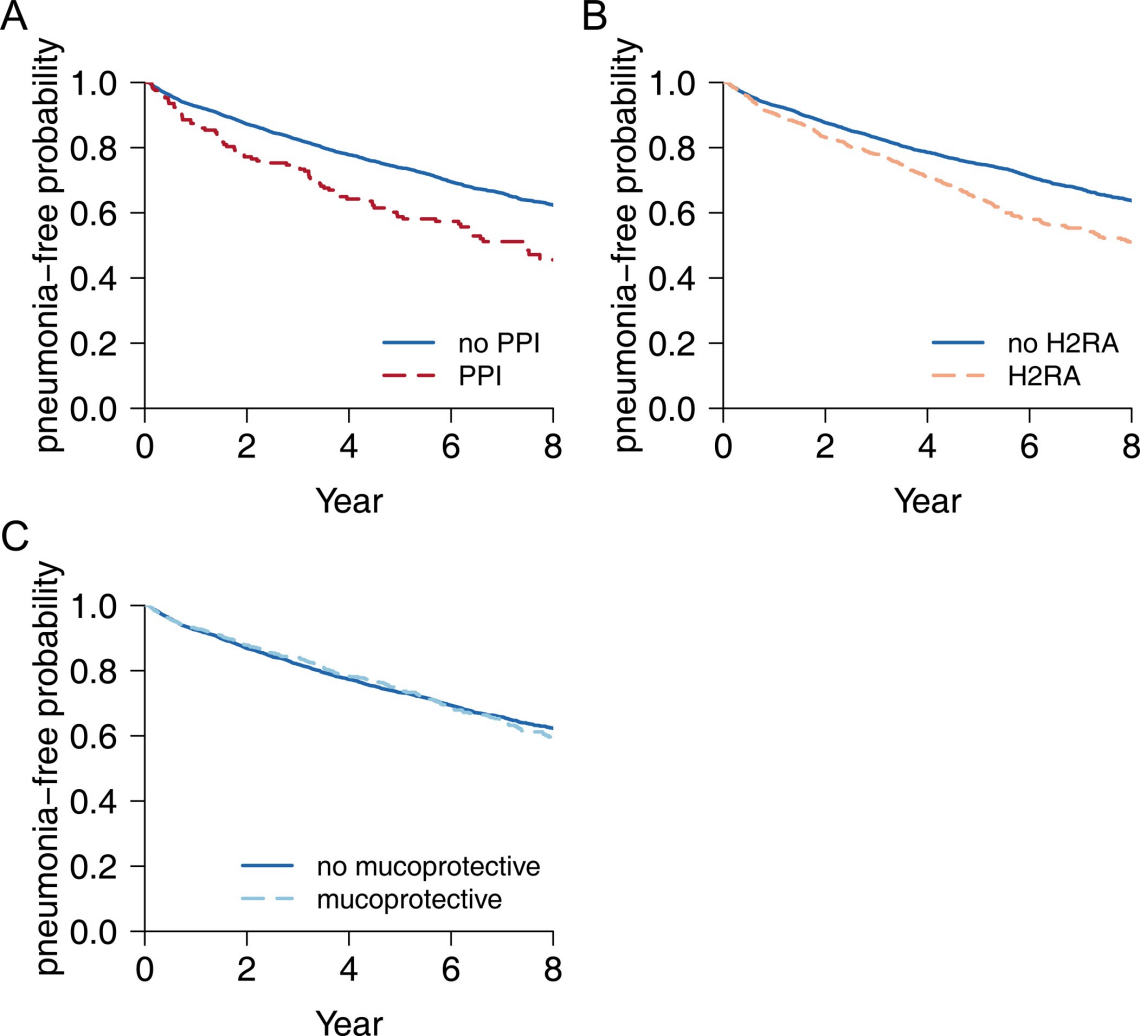
Estimated pneumonia-free probability according to the use of medications during post-stroke follow-up period. Each plot illustrates estimated pneumonia-free survival curves according to the use of PPI (A), H2RA (B), and mucoprotective agents (C) during the followed-up period after stroke. Mucoprotective agents include rebamipide, teprenone, irsogladine, ecabet, polaprezinc, sofalcone, sucralfate, and misoprostol. Abbreviations: H_2_RA, H_2_-receptor antagonist; PPI, proton-pump inhibitor.

During 3.95 ± 3.01 years (mean ± standard deviation) of follow-up, there were 2,035 (24.5%) patients who developed pneumonia after discharge from index stroke. Fig 3 demonstrates the estimated pneumonia-free probability curves considering the use of PPI, H2RA, and mucoprotective agents during follow-up as time-dependent variables. Among the anti-ulcer drugs, treatment with PPI and H2RA was associated with an increased risk for pneumonia (Fig 3A and Fig 3B), but treatment with mucoprotective agents was not (Fig 3C). In the multivariate time-dependent Cox regression model (Table 3), there was a significantly increased risk for pneumonia with use of PPI (adjusted HR [95% CI], 1.56 [1.24–1.96], p<0.001) and H2RA (adjusted HR [95% CI], 1.40 [1.25–1.58], p<0.001). On the other hand, use of mucoprotective agents was not associated with the development of pneumonia (adjusted HR [95% CI], 0.89 [0.78–1.01], p = 0.055). We further analyzed the risk for pneumonia according to the daily dose of PPI, and H2RA (Table 4). There were significant dose-response relationships between risk for pneumonia and daily dose of the anti-acid medications (more pneumonia risk with high dose of PPI and H2RA).

**Table 3:** Effects of medications on the risk for post-stroke pneumonia.

Variable	adjusted HR [95% CI]	P value
**Time-independent variables**		
Sex, male	1.19 [1.09–1.31]	< .001
Age, per 5-year	1.21 [1.18–1.24]	< .001
Hypertension	1.05 [0.94–1.16]	.393
Diabetes mellitus	1.25 [1.13–1.37]	< .001
Myocardial infarction	1.10 [0.93–1.29]	.254
Atrial fibrillation	1.19 [1.02–1.38]	.025
Chronic obstructive pulmonary disease	1.28 [1.15–1.43]	< .001
Thrombolysis (intravenous or intraarterial)	1.03 [0.69–1.54]	.885
Household income		
low	Ref	–
middle	0.96 [0.86–1.07]	.439
high	0.97 [0.87–1.08]	.576
Hospital type		
general hospital	Ref	–
hospital or clinic	1.24 [1.10–1.39]	< .001
Length of hospital stay		
≤17 days	Ref	–
>17 days	1.00 [0.91–1.09]	.986
Year of index stroke		
2002–2005	Ref	–
2006–2009	0.93 [0.84–1.03]	.148
2010–2013	0.93 [0.81–1.07]	.337
**Time-dependent variables**		
Proton pump inhibitor	1.56 [1.24–1.96]	< .001
H_2_-receptor antagonist	1.40 [1.25–1.58]	< .001
Mucoprotective agents*	0.89 [0.78–1.01]	.062

Data were obtained from multivariate time-dependent Cox proportional hazard regression model. Adjustments were made to the variables listed in this table.

*rebamipide, teprenone, irsogladine, ecabet sodium, polaprezinc, sofalcone, sucralfate, and misoprostol.

Abbreviations: HR, hazard ratio; CI, confidence interval.

**Table 4:** Effect of anti-acid medications on pneumonia risk according to the dose intensity.

Medication	Dose intensity	adjusted HR [95% CI]	P value	P value for trend
Proton pump inhibitor	no use	Ref	–	< .001
	low dose	1.34 [0.97–1.85]	.079	
	high dose	1.85 [1.36–2.51]	< .001	
H2-receptor antagonist	no use	Ref	–	< .001
	low dose	1.38 [1.20–1.60]	< .001	
	high dose	1.43 [1.19–1.70]	< .001	

Data are derived by multivariate time-dependent Cox proportional hazard regression model adjusted for variables listed in Table 3.

Abbreviations: HR, hazard ratio; CI, confidence interval.

## Discussion

In this study, we evaluated the risk for pneumonia according to the use of PPI, H2RA, and mucoprotective agents during the long-term follow-up period after acute ischemic stroke using the nation-wide health insurance claims database in Korea. The development of pneumonia after acute ischemic stroke was common; 24.5% of included patients had pneumonia during the mean follow-up period of 3.95 ± 3.01 years. Main finding of this study was that use of the anti-acid medications of PPI and H2RA was significantly associated with the development of post-stroke pneumonia. In contrast to the anti-acid medications, use of mucoprotective agents was not associated with the risk of pneumonia.

For stroke patients, PPI and H2RA are widely subscribed to the control of gastric symptom, prevention and treatment for peptic ulcer and gastrointestinal injury. Ischemic stroke patients generally need life-long use of antiplatelet or anticoagulation for secondary prevention of cardiovascular events. Aspirin is most frequently prescribed antiplatelet, which cause gastric mucosal injury [21]. Concomitant use of non-steroidal anti-inflammatory drugs (NSAID) is also common in patients with cardiovascular risk factors [22]. It is well known that aspirin and NSAID can induce acute and chronic gastroenteropathy; NSAID-induced gastroenteropathy. Aspirin and NSAID inhibit prostaglandin-endoperoxide synthase which reduces the basal protection of cytoprotective prostaglandin E_2_ and prostaglandin I_2_ in the gastric mucosa [23]. Reduced prostaglandins can mediate gastrointestinal injury by low mucosal blood flow, decreased secretion of bicarbonate and mucous, impaired proliferation and repair, and increase of gastric acid and inflammatory molecules such as leukotriene [24]. Prostaglandin independent mucosal injury is also present with topical effects by salicylate or chemical products of NSAID [25]. Clopidogrel, another commonly used antiplatelet, can cause a similar degree of gastric mucosal damage with aspirin [26]. Gastrointestinal bleeding is a serious and potentially life-threatening complication associated with use of antiplatelet and anticoagulation. American Heart Association guidelines for coronary artery disease suggest that use of PPI is reasonable for patients with dual antiplatelet treatment and at increased risk of gastrointestinal bleeding, including those with advanced age and those with concomitant use of warfarin, steroids, or NSAID [27]. For treatment or prevention of NSAID-induced gastroenteropathy, PPI is the recommended treatment of choice [28].

Aside from gastric protection, there is a clinical concern for that PPI and H2RA may increase the development of pneumonia. PPI and H2RA have gastroprotective effects by reducing secretion of gastric acid and acidity in stomach. The acidity of gastric juice is one of primary bactericidal barrier and the rate of killing is strongly dependent to the low pH [29,30]. Considering the biological rationale, bactericidal barrier of gastric acid and anti-acid property of PPI and H2RA, use of the acid suppressants may expose to the risk for pneumonia, especially stroke patients with swallowing difficulty who are highly susceptible to respiratory infections. There were prior studies which reported that risk for pneumonia was increased with the exposure to PPI or H2RA following acute stroke [7–9]. One hospital-based cohort study with 1676 patients admitted for acute stroke reported that exposure to PPI (adjusted odds ratio (OR) [95% CI], 2.7 [1.4–5.4]) and H2RA (adjusted OR [95% CI], 1.6 [0.8–3.4]) during admission were associated with significantly increased risk for hospital-acquired pneumonia [7]. In research with Taiwanese National Health Insurance Research Database, risk of post-stroke pneumonia increased with exposure to H2RA, PPI, or both (adjusted HR; 1.40, 1.38, and 1.57, respectively) [31]. In a hospital based retrospective study including 355 acute stroke patients who could not feed orally for 14 days, the relative risk of H2RA and PPI on pneumonia were 1.24 (95% CI; 0.85–1.81) and 2.00 (95% CI; 1.12–3.57) compared to no usage, respectively [9]. An observational study using the national Japanese Diagnosis Procedure Combination inpatient database showed similar incidence of pneumonia between users of PPI and H2RA after acute stroke [32]. In patients with intracerebral hemorrhage, nosocomial pneumonia was more common in those who received PPI than those did not [8]. In the line with the prior reports of positive relationship between use of acid-suppressive medications and post-stroke pneumonia, we added evidence that use of PPI and H2RA throughout long-term follow-up period were significantly associated with increased risk for post-stroke pneumonia. Our finding of increased risk in proportion to the dose of PPI and H2RA supported the deteriorating effect of the anti-acid medications on the risk for pneumonia. Considering the potential risk for pneumonia, clinician should use caution in prescribing anti-acid medications over the long-term without definite indications. The American Heart Association guidelines for coronary artery disease against the routine use of PPI for patients at low risk of gastrointestinal bleeding without clear indication for the PPI therapy [27].

In this study, there was no increased risk for pneumonia with mucoprotective agents which were also frequently prescribed for control of gastrointestinal symptom or prevention of ulcer. Mucoprotective agents have been used for protection of gastroduodenal mucosa, healing of ulcers, and treatment of gastritis. Without suppression of gastric acid, mucoprotective agents have multiple gastroprotective mechanisms through increase gastric blood flow, prostaglandin biosynthesis and decrease free oxygen radicals [33]. To minimalize the potential risk for post-stroke pneumonia with the anti-acid medications, mucoprotective agents might be safe alternative for gastrointestinal symptom control or mucosal protection to the stroke patients who are susceptible for pneumonia. Compared to anti-acid medications, safety of the mucoprotective agents on pneumonia has been suggested [34]. A meta-analysis and trial sequential analysis of randomized trials showed that sucralfate reduced intensive care unit-acquired pneumonia compared to H2RA in critically ill patients [35]. Sucralfate is one of mucoprotective agents which form a coating over ulcers and provide physical barrier to gastrointestinal tract (mucosal cytoprotectant). Misoprostol is a synthetic prostaglandin E_1_ analogue approved by the Food and Drug Administration for the prevention of drug for prevention of NSAID-associated ulcers. Misoprostol have mucosal protective property by enhancing gastric mucosal blood flow and secretion of mucus and bicarbonate [36]. Multiple clinical trials have demonstrated misoprostol is as effective as PPI for prevention and treatment of gastrointestinal ulcers [37]. Rebamipide is a novel gastroprotective drug which has mechanism of stimulating prostaglandin generation in gastric mucosa, inhibiting neutrophil activation and inflammatory cytokines, and helps in replacement of lost tissue by increasing the expression of epidermal growth factor (EGF) and EGF receptors [38–40]. Rebamipide has anti-oxidative and anti-inflammatory properties which scavenges oxygen-derived free radicals and inhibits the production [41,42]. Randomized trial demonstrated that rebamipide prevented NSAID-induced ulcer as effectively as misoprostol [43]. Rebamipide was equivalent to treatment with lansoprazole in the healing of endoscopic submucosal dissection-induced ulcers [44].

As main strength of our study, use of nation-wide health insurance claims database makes it possible to include > 8000 patients with acute ischemic stroke. Based on the database, we could undergo longitudinal follow-up of the included patients for the development of pneumonia and prescription records. With the strengths, we should acknowledge potential limitations of this study. The risk of developing pneumonia after stroke could be positively related with stroke severity and post stroke disability [45]. Unfortunately, health insurance claims database in Korea did not include data of stroke severity or disability such as National Institutes of Health Stroke Scale or modified Rankin scale. Thus, we could not collect the clinical data of stroke severity or functional disability after stroke, which might be significant prognostic factor for pneumonia. As an alternative, we collected the data for length of hospital stay at index stroke known to be correlated with stroke severity and disability [46, 47]. In the multivariate model adjusted for the length of hospital stay, the association of treatment with PPI and H2RA with increased risk for post-stroke pneumonia remained significant. In addition, we could not collect data of smoking, which might be another important associative factor for pneumonia, because of lack of the data in health insurance database. Due to the nature of retrospective observational design, we could not conclude the causal relationship between the use of medications and risk for pneumonia. There might be uncollected difference in characteristics of patients who received medications and those did not. Because, use of medications were determined based on prescription records, there may be gap between prescription data and individual patient’s actual intake. We defined the development of pneumonia based on to the presence of diagnostic code in NHIS-NSC. Although identification of pneumonia based on ICD-10 code is known to be a reliable, there might be miss-classification of patients who did not visit hospital or by error of registration in clinical practice [13]. Although our study was performed with nationwide cohort, it is difficult to assure that the sample size was adequate to verify the association of pneumonia with anti-ulcer drugs. Further studies are needed to establish the effect of different classes of anti-ulcer drugs on post-stroke pneumonia.

## Conclusions

Treatment with PPI and H2RA is associated with increased risk for post-stroke pneumonia, but treatment with mucoprotective agents is not. Clinician should be aware of the potential impact of anti-acid medications on pneumonia, frequent complication of stroke patients.

## Supporting information

S1 File STROBE checklist cohort. (DOC)
